# Reviewers and awards

**DOI:** 10.1093/ehjdh/ztad076

**Published:** 2023-11-30

**Authors:** Nico Bruining, Peter de Jaegere, Robert van der Boon, Joost Lumens

**Affiliations:** Department of Cardiology, Erasmus MC, Dr Molewaterplein 40, 3015 GD Rotterdam, The Netherlands; Department of Cardiology, Erasmus MC, Dr Molewaterplein 40, 3015 GD Rotterdam, The Netherlands; Department of Cardiology, Erasmus MC, Dr Molewaterplein 40, 3015 GD Rotterdam, The Netherlands; CARIM School for Cardiovascular Diseases, Maastricht University Medical Center, Maastricht, The Netherlands

## Reviewers and recognition

The success of the *European Heart Journal – Digital Health* (EHJ-DH) is owed to both the submitting authors and those behind the scenes, our highly valued reviewers. Their dedicated contributions are crucial. The Editors acknowledge the challenge of finding time in our busy schedules for quality peer reviews and extend profound gratitude to each of you for generously sharing your invaluable expertise. Through this collective effort, the scientific quality of numerous manuscripts has significantly improved.

This collaborative endeavour resulted in our successful indexation in Pubmed and Pubmed Central in early 2023. This milestone not only contributes to the journal’s growth and expansion but also aligns with our ambitions, including the goal of becoming the leading journal in *Cardiovascular Digital Health*.

**Figure 1:**
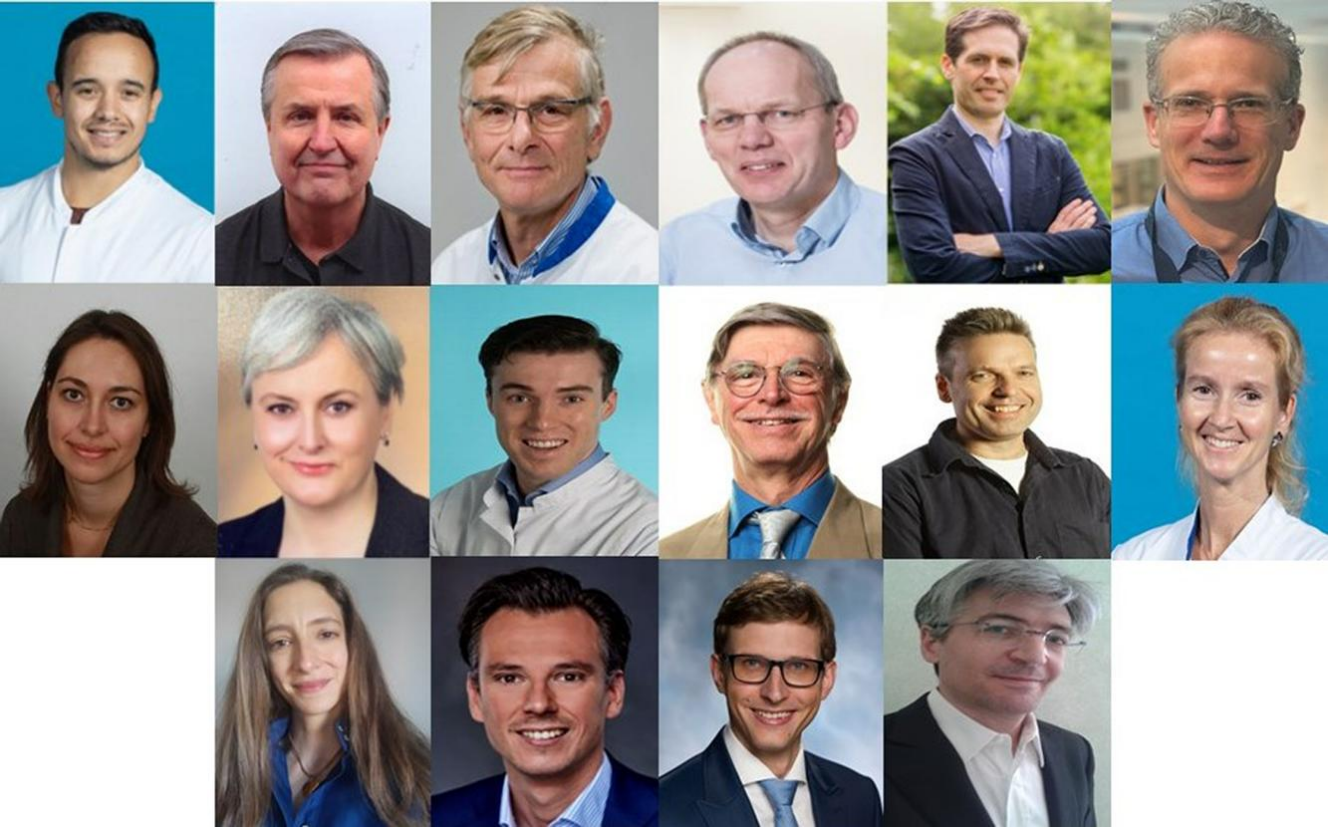
The Top Reviewers with Robert van der Boon as the absolute top reviewer at the top row on the left. Further on the top row from left to right, Enno van der Velde, Peter de Jaegere, Eric Boersma, Ruben Casado-Arroyo, and Rogier Barendse. On the middle row, Isabella Kardys, Nurgül Keser, Roderick Treskes, Riccardo Asteggiano, Peter van Dam, and Natasja de Groot. At the bottom, Joanna Ribeiro, Mark Schuuring, Marton Tokodi, and Enrico Caiani.

In the past year, 215 reviewers played a pivotal role in evaluating the manuscripts we received. Special acknowledgment goes to our Top Reviewers, with Dr Robert van der Boon standing out as the absolute top reviewer (*Figure 1*).

In the table, in alphabetical order, are the names of all our reviewers for the *European Heart Journal – Digital Health* (*Table 1*). In case we have unintentionally omitted someone due to an unfortunate mistake, please accept our apologies. We owe a great debt of gratitude to all of you! Thank you so much, and we look forward to continuing working with you and hope to receive your work as well!

**Table 1:** 

Last name	First name
Adedinsewo	Demilade
Agostoni	Piergiuseppe
Ahmed	Nida
Akbilgic	Oguz
Alexander	Thomas
Alsharqi	Maryam
Attia	Zachi I
Avram	Robert
Baart	Sara
Babayiğit	Erdi
Baldi	Enrico
Bartuś	Stanisław
Batalik	Ladislav
Bauer	Axel
Beatty	Alexis
Beerten	Simon
Ben Gal	Tuvia
Bertrand	Philippe
Bhattacharyya	Anirban
Biondi Zoccai	G
Boriani	Giuseppe
Brittain	Evan
Brown	Emily
Bruining	Nico
Brunner-La Rocca	Hans-Peter
Caliskan	Kadir
Camm	John
Capelli	Claudio
Casado-Arroyo	Ruben
Casiglia	Edoardo
Cazenave Gassiot	Amaury
Chan	Kei Hang Katie
Ching	Chi Keong
Cosyns	Bernard
Cummins	Paul
Dörr	Marcus
Dürschmied	Daniel
Daemen	Joost
De Biase	Luciano
de Groot	Natasja
den Ruijter	Hester
Dijkstra	Jouke
Dilaveris	Polychronis
Dominguez	Helena
Dou	Kefei
Duchateau	Nicolas
Dunias	Zoe
Fabritz	Larissa
Fahed	Akl
Farjo	Peter
Fraser	Alan
Frisch	Daniel
Fyenbo	Daniel
Gao	Tianxin
García-Escobar	Artemio
Garcia-Garcia	Hector
Ghisi	Gabriela Lima de Melo
Gillebert	Thierry
Goldsweig	Andrew
Goto	Shinya
Goto	Shinya
Grégoire	Jean-Marie
Grenne	B
Gu	Yuan
Gu	Sophie
Guckert	Michael
Hashim	Hashim Talib
Haynes	Sarah
Heida	Harmen
Hemels	Martin
Hendriks	Jeroen
Hida	Satoshi
Himmelreich	Jelle
Howard	James
Huberts	Wouter
Hung	Chung-Lieh
Islam	Sheikh Mohammed Shariful
Jung	Christian
Kaese	Sven
Kaji	Shuichiro
Kario	Kazuomi
Kelm	Malte
Khera	Rohan
Knackstedt	Christian
Knott	Kristopher
Kodera	Satoshi
Koifman	Edward
Kolk	Maarten
Kolli	Kranthi K
Kovács	Attila
Krzesiński	Paweł
Kwan	Jennifer
Kwon	Joon-myoung
Ladeiras Lopes	Ricardo
Lamata	Pablo
Lanzer	Peter
Lear	Scott A.
Lemos	Pedro
Lenzen	Mattie
Leone	Antonio Maria
Li	Ying-Chun
Lim	Daniel
Lindman	Brian
Lingsma	Hester
Lip	Gregory
Loewe	Axel
Love	Sharon B.
Luehr	Maximilian
Luo	Hongxing
Lurz	Philipp
Münzel	Thomas
MacRae	Calum
Mahmoodi	B
Mahtab	Edris
Makimoto	Hisaki
Malik	Marek
mamprin	M
Marceglia	Sara
Margulies	Ken
Marrouche	Nassir
Masud	Jakir Hossain Bhuiyan
Mengxing	Liu
Meyers	Harvey
Michiels	Kilian
Mihailidou	Anastasia
Mital	Seema
Mittal	Suneet
Mohammadnia	Niekbachsh
Molenaar	Mitchel
Moons	Philip
Mortazavi	Bobak J.
Mueller	Stephan
Muhammad	Noryanti
Nakayama	Masafumi
Nasir	Khurram
Neubeck	Lis
Nonaka	Naoki
Norekval	TM
Oikonomou	Evangelos
Okumura	Yasuo
Olier	Ivan
Ong	Peter
Ozdemir	Mehmet Akif
Pellegrini	Dario
Penso	M
Pepera	Garyfallia
Peters	S
Peters	Nicholas
Peyster	Eliot G
Piazza	Nicolo
Platonov	Pyotr
Puyol-Antón	Esther
Ramirez	Julia
Redfern	Julie
Redfern	Oliver
Reps	Jenna Marie
Roselló-Lletí	Esther Roselló
Saber	Amir
Saner	Hugo
Sarajlic	Philip
Schlegel	Todd T.
Schmidt	Samuel
Scholte	Niels
Scoccia	A
Selder	Jasper
Seligman	Henry
Shandhi	Md Mobashir Hasan
Sicari	Rosa
Siontis	Konstantinos
Smith	Stephen
Soejima	Kyoko
Soulaidopoulos	Stergios
Stöllberger	Claudia
Steijlen	A.S.M.
Strauss	Bradley
Stub	Dion
Suh	Jung-Won
Sun	Jiangming
Sung	Jidong
Suri	Jasjit
Svennberg	Emma
Szymanski	Mariusz
Taddei	Stefano
Takahashi	Kengo
Takaoka	Hiroyuki
Tekkeşin	Ahmet İlker
Tilz	Roland R.
Tokodi	Márton
Tonino	Pim
Trask	Aaron
Trinkley	Katy
Tsang	Wendy
Väliaho	Eemu-Samuli
Vaidya	Ajay
van de Leur	Rutger
van der Velde	Enno
van Es	Rene
Van Heuverswyn	Frederic
van Smeden	Maarten
Van Spall	Harriette
Velasco	Juan
Viljoen	Charle
Visseren	Frank
Volgman	Annabelle
Wang	Cong
Wiegman	Albert
Winkel	Bo Gregers
Xue	Li
Yagil	Avi
Yang	Gang
Yang	Min
Yu	Bo
Zei	Paul
Zhou	Yujie

Sincerely yours, Nico Bruining, Peter de Jaegere, Robert van der Boon, and Joost Lumens.

